# Sphingolipids as cell fate regulators in lung development and disease

**DOI:** 10.1007/s10495-015-1112-6

**Published:** 2015-03-10

**Authors:** Joyce Lee, Behzad Yeganeh, Leonardo Ermini, Martin Post

**Affiliations:** 1 Program in Physiology and Experimental Medicine, Peter Gilgan Centre for Research and Learning, The Hospital for Sick Children, Toronto, ON M5G 0A4 Canada; 2 Institute of Medical Science, University of Toronto, Toronto, ON Canada

**Keywords:** Ceramide, Sphingosine, Apoptosis, Caspase, Autophagy, Necroptosis, Lung development, Lung diseases

## Abstract

Sphingolipids are a diverse class of signaling molecules implicated in many important aspects of cellular biology, including growth, differentiation, apoptosis, and autophagy. Autophagy and apoptosis are fundamental physiological processes essential for the maintenance of cellular and tissue homeostasis. There is great interest into the investigation of sphingolipids and their roles in regulating these key physiological processes as well as the manifestation of several disease states. With what is known to date, the entire scope of sphingolipid signaling is too broad, and a single review would hardly scratch the surface. Therefore, this review attempts to highlight the significance of sphingolipids in determining cell fate (e.g. apoptosis, autophagy, cell survival) in the context of the healthy lung, as well as various respiratory diseases including acute lung injury, acute respiratory distress syndrome, bronchopulmonary dysplasia, asthma, chronic obstructive pulmonary disease, emphysema, and cystic fibrosis. We present an overview of the latest findings related to sphingolipids and their metabolites, provide a short introduction to autophagy and apoptosis, and then briefly highlight the regulatory roles of sphingolipid metabolites in switching between cell survival and cell death. Finally, we describe functions of sphingolipids in autophagy and apoptosis in lung homeostasis, especially in the context of the aforementioned diseases.

## Introduction

**Fig. 1:**
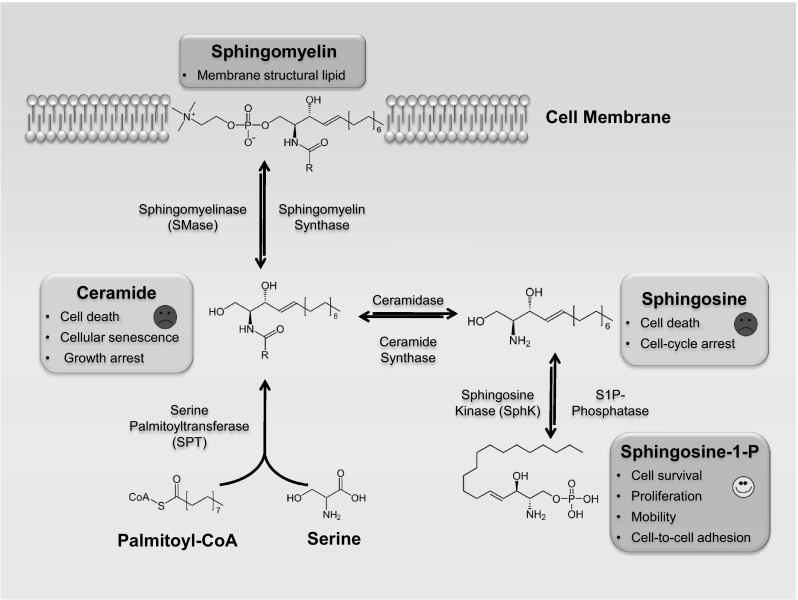
*Overview of sphingolipid metabolism and their major functions*. Refer to text for further details. *CoA* coenzyme A, *S1P* sphingosine-1-phosphate, *SMase* sphingomyelinase, *SphK* sphingosine kinase, *SPT* serine palmitoyltransferase

Sphingolipids are a class of lipids that were named after the mythological Sphinx because of their enigmatic nature. They were initially thought to serve strictly as structural components of the membrane bilayer, but have now been implicated in various cell-signaling pathways including cell proliferation, differentiation, and programmed cell death (PCD) [1]. These molecules display amphiphatic properties in which the hydrophobic end is comprised of a sphingoid base that is linked to a fatty acid, while the hydrophilic end varies in structures consisting of hydroxyl groups, phosphates and sugar residues. Different lengths, saturations, and hydroxylations of fatty acids, as well as different head groups result in an immense diversity of the sphingolipids species [2]. All sphingolipids can be generated from a derivation of ceramide. For example, as it is shown in Fig. 1, ceramide can be deacylated by ceramidase to sphingosine that then can be phosphorylated by a sphingosine kinase isoenzyme (SphK_1_ or SphK_2_) to form sphingosine-1-phosphate (S1P) [3, 4]. Alternatively, sphingosine can be acylated by ceramide synthase to give rise to ceramide. Both ceramide and sphingosine can act as a second messenger to promote apoptosis, cellular senescence, and growth arrest [5]. Sphingomyelin synthase converts ceramide to sphingomyelin, a structural lipid mainly localized to the outer membrane leaflet, while sphingomyelin can give rise to ceramide by the action of sphingomyelinase (SMase) isoenzymes [6]. Ceramide and S1P have received attention as they appear to play opposing roles in a dynamic relationship known as the “sphingolipid rheostat” [1, 7, 8]. At one end of the scale, ceramide is typically recognized to initiate apoptosis and growth arrest, whereas S1P, at the other end, promotes cell proliferation, survival, mobility, and cell-to-cell adhesion [9–11]. These two sphingolipids have been shown to play a role in cell fate processes such as apoptosis, and more recently, autophagy. A general overview of sphingolipid metabolism and their major functions is shown in Fig. 1. The role of sphingolipids in lung cell fate will be explored in this review.

### Ceramide

Ceramide can be generated through three known pathways. De novo synthesis of ceramide is characterized by the rate-limiting step of condensation between serine and palmitoyl-CoA catalyzed by serine palmitoyltransferase (SPT) [12]. Any of the sphingomyelinase isoenzymes, acid sphingomyelinase (aSMase), neutral sphingomyelinase (nSMase), and alkaline sphingomyelinase can use sphingomyelin as a substrate to produce ceramide [13]. Finally, synthesis of ceramide through a recycling loop from sphingosine and glycosphingolipids can also occur by the reverse activity of ceramidase [14].

Ceramide is a well-known critical mediator of various cell death pathways, including apoptosis and necrosis [15, 16]. Increased ceramide levels have been associated with apoptotic cell death in both homeostatic systems as well as pathological settings as a result of cellular insults including oxidative stress, chemotherapeutic agents, ischemia and radiation [5, 17–20]. Studies investigating the mechanism of ceramide-mediated apoptosis have demonstrated that ceramide can act on both the intrinsic (mitochondrial) and extrinsic pathways of apoptosis in a context-dependent manner [21, 22]. Moreover, ceramide is able to induce apoptosis by recruitment of death receptors to lipid rafts and assembly of channels in the outer membrane of the mitochondria promoting the release of cytochrome *c,* only to mention a few amongst several investigated pathways [22–24]. It has more recently been shown that ceramide has a significant impact on autophagy, influencing cellular fate under stress conditions such as amino acid deprivation, mitochondrial damage, and ER stress [25–30].

### Sphingosine-1-phosphate

S1P is well recognized to play critical roles in not only cell proliferation and survival, but also in cell mobility and chemotaxis, cell-to-cell adhesion, angiogenesis, intracellular calcium homeostasis, and cytoskeletal organization [9, 31–33]. Synthesis of S1P can occur by the hydrolysis of sphingolipids from the plasma membrane into ceramide and subsequent *N*-deacetylation of ceramide to form sphingosine that can be phosphorylated by SphK_1_ or SphK_2_ [3, 4] to generate S1P. Signaling actions of S1P can be executed within the intracellular space, as well as the extracellular space when it acts as a ligand of five G-protein coupled receptors (S1P_1_–S1P_5_) [34, 35]. These receptors allow S1P to influence signaling processes including angiogenesis, heart development, immunity, and cell movement depending on the receptor that is ligated, cell type, and the cellular context [3]. S1P is currently understood to promote cell survival by inhibition of enzymes involved in ceramide synthesis, as well as activation of the nuclear factor-κB (NF-κB) signaling pathway [36–39]. Furthermore, S1P has also been recently suggested to induce autophagy accordingly with its protective role against apoptosis [7, 40, 41].

The broad effects of second messengers generated through metabolism of sphingolipids (ceramide, S1P) on regulation of cell fate as well as the plasticity of sphingolipid metabolism suggests a variety of possible mechanisms for controlling cell fate. Further study will be required to elucidate the underlying mechanisms regulating cell survival or death decisions by these family members.

## Determination of cell fate

### Apoptosis

PCD is an essential physiological process involved in development, aging, and tissue homeostasis which maintains normal cellular fate in different organisms [42]. Apoptosis (PCD1) is a widely recognized mode of PCD in which complex molecular signaling systems trigger an orderly, energy-dependent enzymatic breakdown of DNA, lipids, and other macromolecules [43]. Cells undergoing apoptosis show typical, well-defined morphological changes characterized as rounding of the cell, shrinkage of pseudopods, decreased cellular volume, chromatin condensation (pyknosis), nuclear fragmentation (karyorrhexis) along with little or no ultrastructural reformations of organelles in the cytoplasm followed by plasma membrane blebbing, and ingestion by phagocytes [43, 44].

In contrast to necrosis, apoptosis does not induce inflammation since apoptotic cells do not release their cellular contents into the surrounding interstitial tissue and rather are quickly engulfed by macrophages or adjacent normal cells [45, 46]. The mechanism of apoptosis is highly synchronized and is coordinated by an extensively studied group of cysteine proteases known as cysteine aspartate-specific proteases (caspases). Caspases are widely expressed in an inactive proenzyme form (procaspases), localized in the nucleus, cytoplasm, endoplasmic reticulum (ER), the mitochondrial intermediate space, and can be translocated to the plasma membrane [43]. Caspases have proteolytic activity and are able to cleave proteins at an internal aspartic acid site, although different caspases have specificities involving recognition of neighboring amino acids [47, 48]. According to their order of activation, caspases are classified into two groups: (1) the initiator caspases (i.e. caspase-2, -8, -9, and -10), (2) and effector caspases (i.e. caspase-3, -6, and -7) [49, 50]. Once activated, caspases can often activate other procaspases, allowing initiation of a protease cascade that leads to an irreversible commitment towards cell death.

**Fig. 2:**
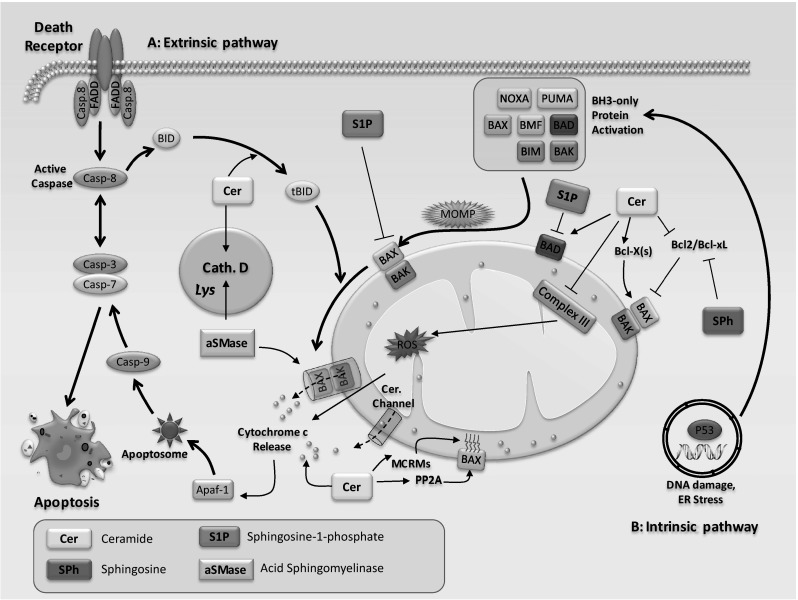
*Schematic representation of key events in the apoptotic pathway and regulation of apoptosis by sphingolipids*. There are two main apoptotic pathways. **A** Extrinsic pathway is triggered when cell death ligands (e.g., FasL, APO-2L, TRAIL, TNF) bind to their respective death-receptors (e.g., Fas, DR4, DR5, TNF-R1) and initiates pro-caspase-8 activation by recruiting FADD. Activation of caspase-8 results in cleavage of effector caspases, such as caspase-3,-6,-7, which are involved in the core apoptosis pathway. Furthermore, caspase-8 can truncate BID (tBID), which later induces the intrinsic pathway. **B** The intrinsic pathway can be directly initiated by a variety of stress signals. Stress signals initiate DNA damage and p53 phosphorylation, which leads to the up-regulation of BH3 only proteins and consequently results in mitochondrial translocation and oligomerization of BAX/BAK, followed by MOMP. Mitochondrial damage leads to cytochrome *c* release into the cytoplasm. Cytosolic cytochrome *c* binds to the pro-apoptotic factor Apaf-1 (in the presence of dATP) to form an apoptosome. Apoptosomes then activate caspase-9, which later leads to the activation of caspases-3 and-7, and subsequently to nuclear fragmentation and also chromatin condensation. Sphingolipids have been shown to modulate apoptosis at multiple steps of the process. Sphingolipids may directly affect mitochondria, a strategic center in the control of apoptosis. Ceramide forms channels in mitochondrial outer membranes and promotes the release of cytochrome *c* for caspase-9 activation. Ceramide channel formation has also shown to be inhibited by dihydroceramide. Furthermore, ceramide generates reactive oxygen species (ROS) via inhibition of mitochondrial complex III. The apoptotic action of ceramide could also be mediated by the recruitment and activation of pro-apoptotic Bax at the mitochondria through the PP2A-dependent dephosphorylation of Bax and formation of mitochondrial ceramide-rich macrodomains (MCRMs). aSMase-released ceramide binds directly to lysosomal protease cathepsin D, leading to cathepsin D activation, resulting in cleavage of the BH3-only protein BID and induction of the mitochondrial pathway of apoptosis. Sphingosine has been shown to downregulate expression of anti-apoptotic proteins, Bcl-2 and Bcl-xL, to enhance apoptosis. While ceramide-mediated activation of pro-apoptotic protein, BAD, promotes apoptosis, S1P suppresses apoptosis via BAD inactivation

There are at least two major pathways in mammals that are involved in the initiation of apoptosis, namely the extrinsic pathway and the intrinsic pathway (Fig. 2). Initiator caspases are activated upon extrinsic or intrinsic stimuli that lead to the activation of executioner caspases [49, 50]. Extrinsic and intrinsic pathways differ in their induction and regulation, although there is now evidence that the two pathways are linked and intersect at different stages where molecules in one pathway can influence the other [51].

The extrinsic pathway, also commonly referred to as the death receptor pathway, is initiated through the ligation of death receptors (Fas, DR4, DR5, TNF-R1) by their specific ligands (e.g., FasL, APO-2L, TRAIL, and TNF) [52]. Interaction of a death ligand to its corresponding receptor leads to activation of initiator caspase-8. Active caspase-8 can affect the mitochondria via truncated BID (tBID) and causes mitochondrial initiator caspase (caspase-9) activation [43, 53]. All of these events culminate to effector caspase activation (caspase-3, -7, -6) [52], resulting in cleavage of different substrates like cytokeratins, PARP, plasma membrane cytoskeletal proteins (alpha fodrin), and subsequently provokes morphological and biochemical aspects of apoptosis.

Sphingolipids have been shown to have direct effects on regulators of the extrinsic pathway of apoptosis (Fig. 2). For instance, ceramide has been reported to activate protein kinase Cζ (PKCζ), which regulates the activation of c-Jun NH2-terminal kinase 1 (JNK1) and inhibition of protein kinase B (PKB or Akt) to induce apoptosis [54–57]. Ceramides are also able to directly bind and activate the lysosomal protease cathepsin D, a direct effector of apoptosis [58, 59]. Upon stimulation with tumor necrosis factor (TNF)-α increased levels of ceramide stimulate cathepsin D-mediated cleavage of BID to activate the apoptotic pathway [58, 59]. Sphingosine acts also as a pro-apoptotic signaling lipid via suppression of the MAPK/ERK signaling pathway [60]. In contrast, S1P is a suppressor of ceramide-mediated activation of JNK1 by activating pro-survival Akt/mTOR complex 1 (mTORC1), MAPK/ERK, and NF-κB signaling pathways [33]. Thus, regulators of the extrinsic pathway of apoptosis are affected in response to various sphingolipids (Fig. 2).

The intrinsic or mitochondrial pathway can be initiated following ER stress, exposure to stresses such as cytotoxic drugs, ultraviolet radiation, and free radicals which cause DNA damage [61–64]. DNA damage and ER stress activates pro-apoptotic members of the Bcl-2 family (Bax/Bak) and induces mitochondrial outer membrane permeabilization (MOMP), ultimately leading to caspase-dependent or independent apoptosis [52, 65]. Anti-apoptotic Bcl-2 proteins (Bcl-2 and Bcl-xL) counteracts pro-apoptotic proteins and can delay or inhibit apoptosis [66]. Following MOMP, release of various polypeptides such as cytochrome *c* from the mitochondrial intermembrane space promotes caspase activation and apoptosis. Cytochrome *c* in the cytosol binds with apaf-1 (apoptotic protease-activating factor-1), inducing its oligomerization and thereby forming a structure termed the apoptosome. Formation of the death-inducing signaling complex (DISC) and apoptosome results in activation of initiator caspases 8 and 9, respectively [7, 67]. Each then activates effector caspase-3, which ultimately results in hallmark apoptotic morphological signatures such as loss of cytoplasm, blebbing of the plasma membrane, and fragmentation of DNA in the nucleus [67, 68].

Several studies have highlighted the regulatory roles of sphingolipids on the intrinsic pathway of apoptosis (Fig. 2). Ceramides promote the intrinsic pathway by formation of channels in the outer membrane of the mitochondria to promote the release of cytochrome *c* resulting in caspase-9 activation [22, 23]. On the other hand, dihydroceramide has been shown to inhibit ceramide channel formation [69]. In addition, the intrinsic pathway can also be stimulated by the inhibitory action of ceramide on mitochondrial complex III to generate reactive oxygen species (ROS) [70, 71]. The apoptotic action of ceramide can also be mediated by the recruitment and activation of pro-apoptotic Bax at the mitochondria through the PP2A-dependent dephosphorylation of Bax and formation of mitochondrial ceramide-rich macrodomains (MCRMs) [72, 73]. Intriguingly, ceramides synthesized in the ER have been shown to transport to the mitochondria where they transiently permeabilize its outer membrane and stimulate the release of cytochrome *c* [74]. Such exchange may limit the need for ceramide synthesis in the mitochondria to reach ceramide levels required for initiation of the intrinsic apoptotic pathway [74]. It has been shown that aSMase-released ceramide is able to bind directly to lysosomal protease cathepsin D, leading to cathepsin D activation. The activated cathepsin D subsequently cleaves BH3-only protein BID and promotes the mitochondrial apoptotic pathway [58]. Furthermore, while ceramide-mediated activation of pro-apoptotic protein, BAD, promotes apoptosis, S1P suppresses apoptosis via BAD inactivation [75–77]. Sphingosine has been shown to downregulate expression of anti-apoptotic proteins Bcl-2 and Bcl-xL to enhance apoptosis [78, 79].

A far less well understood form of PCD, termed programmed necrosis or necroptosis, has emerged that does not appear to require caspase activity and is morphologically distinct from apoptosis. Necroptosis is an alternative receptor interacting protein (RIP)-mediated form of cell death, which is dependent on TNF receptor, Fas and TNF-related apoptosis-inducing ligand (TRAIL) receptor activation [80–82]. At a biochemical level, while necroptosis displays molecular pathways distinct from those controlling cell deaths by apoptosis or autophagy and represent a different morphology, significant cross-talk between these pathways have been described in different studies [83–85]. In some cases, molecular pathways of necroptosis may involve cellular metabolic alterations associated with mitochondria and the overproduction of ROS [86, 87] via different macromolecules such as ceramide. For instance, Ardestani et al. [87] demonstrated that the mTNF-α isoform is an effective inducer of programmed necrosis through a caspase independent ceramide-induced ROS pathway in mouse fibroblast (L929) cells. In another study with L929 cells, it has been shown that docosahexaenoic acid antagonized TNF-α-induced necroptosis through attenuating ROS generation, ceramide production and lysosomal dysfunction [88]. However, other studies using human intestinal epithelial (HT-29) cells and human monocytic (U937) cells demonstrated that ROS are not required for necroptosis [89, 90]. These disparate findings show the limitation of in vitro bioassays for the evaluation of necroptosis signaling; hence, the exact role of ceramide and whether or not necroptosis requires ROS or mitochondria has yet to be fully determined in vivo.

### Autophagy

Autophagy is a tightly regulated catabolic process that supplies energy during development and in response to nutrient stress by carrying out lysosomal degradation of cell contents [91]. Despite its major role as a survival mechanism, autophagy has previously been classified as PCD2 based on morphological grounds, termed “autophagic cell death” to describe a form of caspase-independent necrosis-like cell death associated with the accumulation of autophagosomes in cells [92]. The existence of autophagic cell death as a bona fide death process is still controversial, and the casual relationship between autophagy and cell death remains unproven [93, 94]. Nevertheless, many studies have pointed to intimate relationships between autophagy and cellular death programs, which are not yet fully understood [95].

The autophagy pathway is evolutionarily conserved from early eukaryotes to mammals with as many as 38 Autophagy Related Genes (*ATG*) identified in yeasts and their human orthologs [96]. Autophagy is divided into three distinct forms: chaperone-mediated autophagy (CMA), microautophagy and macroautophagy [97]. A variety of stress stimuli including long term starvation, exposure to cytotoxic compounds, or oxidative stress can lead to CMA activation which selectively degrades cytosolic proteins in lysosomes [98]. The exact molecular mechanism that triggers microautophagy remains unknown. However, guanosine-5′-triphosphate (GTP) hydrolysis and calcium ions are considered as major initiators of this event in yeast [99]. Macroautophagy (referred to here as autophagy) degrades the bulk of damaged cytoplasmic organelles and proteins. Autophagy includes mitophagy (mitochondrial autophagy), ribophagy (ribosomal autophagy), pexophagy (peroxisome autophagy), ER-phagy (endoplasmic reticulum autophagy), aggrephagy (protein aggregate autophagy) and lipophagy (fat autophagy) [97].

**Fig. 3:**
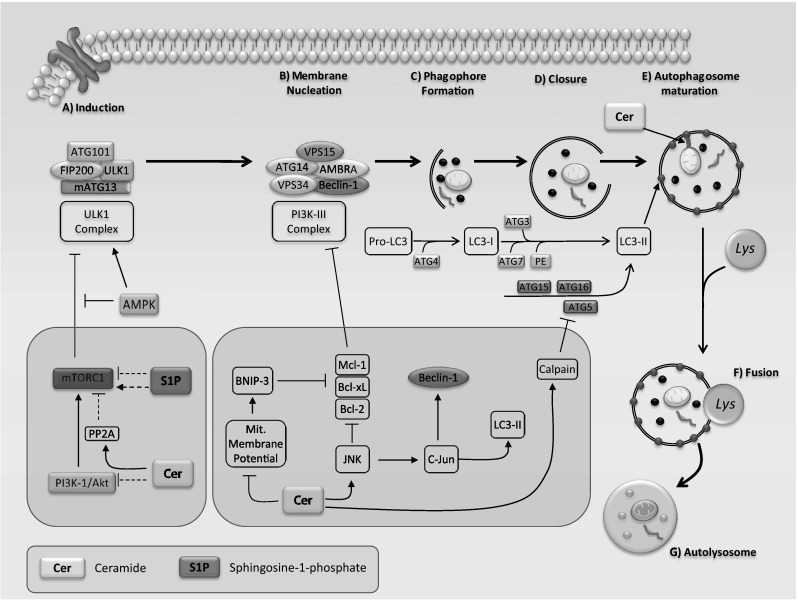
*A schematic overview of autophagy machinery and its regulation by sphingolipids*. **A** *Autophagy induction and nucleation of phagophore membranes (pre-autophagosomal structures)*: in nutrient rich conditions (insulin, growth factors and amino acids), the mTORC1 kinase associates with the ULK1 complex to inhibit the initiation of autophagy. However, under growth factor deprivation or nutrient starvation, energy sensor AMPK activates the ULK1 complex by directly phosphorylating ULK1 and by suppression of mTORC1 activity through phosphorylation, and initiates vesicle nucleation. Phosphorylated and active ULK1 also promotes phosphorylation of Atg13 and FIP200, and dissociates from mTORC1. PI3K-III and VPS34 together with ATG14, AMBRA1, VPS15, and Beclin-1 form a protein complex (PI3K-III complex) and initiates phagophore formation. *Autophagosome formation and maturation*: Two ubiquitin-like proteins, Atg12 and LC3, are involved in double-membrane vesicle (autophagosome) formation, elongation, and closure. Atg12 is conjugated with Atg5 by Atg7 and Atg10, which then form a complex with Atg16. This complex works with Atg7 and Atg3 to conjugate LC3-I with the polar head of phosphatidylethanolamine (PE) to produce LC3-II, which is specifically located on autophagosome structures. Autophagosomes are sequentially fused with lysosomes to form autolysosomes. In the autolysosome, the autophagosomal cargoes are digested by lysosomal hydrolases and the contents are released for metabolic recycling. **B** Sphingolipids have direct effects on different stages of the autophagy pathway. At the initial step, ceramide may stimulate autophagy via PI3K-1/Akt activation which suppresses the inhibitory effects of mTORC1 on autophagy. Acid sphingomyelinase-derived ceramide also increases autophagy via reducing mTORC1 activity during amino acid deprivation in a PP2A-dependent manner. C2-ceramide-induced decrease of mitochondrial membrane potential upregulates BNIP3 expression which ultimately leads to induction of autophagy through dissociation of Beclin-1 from Bcl-2, Bcl-xL, and Mcl-1 in a competitive manner. Similarly, ceramide-mediated activation of JNK disrupts the inhibitory Beclin-1:Bcl-2 complex through direct phosphorylation of Bcl-2. Furthermore, ceramide-mediated activation of transcription factor c-Jun may increase autophagy activation via upregulation of Beclin-1 and LC3 expression. Ceramide may also activate calpain which subsequently cleaves Atg5 to generate a protein fragment that leads to suppression of autophagy and apoptosis induction. Mitochondrial ceramide has been shown to mediate mitophagy through the direct interaction between ceramide and LC3-II

Autophagosomes are the major particles that are formed and processed during the autophagy pathway. An autophagosome includes a double-membrane vesicle destined for degradation of proteins and organelles (cargo) which finally fuse with lysosomes to form autophagolysosomes [100]. Autophagosome formation requires the expression of *ATG* genes which control levels of Atg proteins [101]. Formation of the structures occurs via three main steps: (1) initiation (induction), (2) elongation, and (3) maturation (closure), with subsequent fusion with lysosomes to form the autolysosome or amphisome [97] (Fig. 3).

The mammalian target of rapamycin (mTOR) constitutes a central checkpoint that suppresses autophagy via restraining the kinase activity of UNC-51-like kinase (ULK1) [102, 103]. mTORC1 contains the mTOR catalytic subunit (raptor/GβL/PRAS40/deptor) and phosphorylates ULK1 in the absence of amino acid and growth factor signals [104]. ULK1 Ser/Thr protein kinase, Atg13, and FIP200 (FIP200 is the mammalian homolog of the yeast Atg17) form the ULK1 complex [105–107] and regulates autophagy by phosphorylation of Atg13 and FIP200 [108]. Under stresses such as nutrient starvation, mTORC1 is inhibited by AMP-activated protein kinase (AMPK) resulting in the disassociation of ULK1 and Atg 13. The ULK1 complex is then phosphorylated by AMPK and then initiates vesicle nucleation [105].

Beclin-1 is a platform protein and its complex with class III phosphatidylinositol 3-kinase (PI3K) has a key regulatory role in nucleation and assembly of the initial phagophore membrane [109, 110]. Beclin-1 associates with Vps15, Vps34, and Ambra1 to form a complex that regulates class III PI3K, which forms phosphatidylinositol-3-phosphate (PI3P). Furthermore, Beclin-1 complex can also trigger autophagy via JNK1, and death-associated protein kinase (DAPK) [111]. PI3P is needed for recruitment of other Atg proteins as well as formation of the Ω-shape of initial vesicle nucleation which can be sourced from the outer mitochondrial membrane, ER and/or the plasma membrane [112, 113]. Elongation of the vesicle requires two ubiquitin-like conjugation systems. The first involves the formation of the Atg12–Atg5 conjugate, which appears to act as the E3 ligase of microtubule-associated protein 1 light chain 3 (LC3). The second system initiates autophagosome formation and involves the conjugation of LC3 to the polar head of phosphatidylethanolamine (PE) by release of Atg8 from Atg3 [97]. This conjugation leads to the conversion of the soluble LC3 (LC3-I) to its lipidated form, LC3-II, which is incorporated into the autophagosome membrane [113, 114]. Atg9 has also been shown to be recruited by the Atg1-Atg13 signaling complex and has an important role in expansion of the autophagosome precursor [115]. The conversion of LC3 from LC3-I to LC3-II is regarded as a critical step in autophagosome formation [116] and also represents a hallmark for detecting autophagy [97, 116]. Following elongation, expansion, and closure, the autophagosome with cytoplasmic material finally fuses with the lysosome forming an autophagolysosome, and its contents are subsequently digested by lysosomal enzymes [97].

When the autophagosome proceeds to fuse with a lysosome, its vesicular contents are degraded into macromolecules and are recycled back to the cytosol [7, 91, 112]. This process is conserved in all eukaryotic cells and serves to maintain homeostasis under normal conditions to prevent accumulation of excess/damaged organelles and proteins. However, under conditions of stress such as nutrient starvation, oxidative stress, pathogen infection or hypoxia, autophagy serves as an adaptive cell survival response that could also lead to cell death in situations of defective or excessive autophagy [103, 112].

The regulation of autophagy or apoptosis and the interplay between these two pathways is a complex process involving various key regulators. Among them, sphingolipids have been shown to have significant roles in different organ systems [117–121]. For example, ceramide and S1P have both been demonstrated to induce autophagy by various mechanisms. Because of the paradoxical roles of autophagy, ceramide-dependent autophagy could either promote cell death (by inducing autophagy cell death, or “switching” the cell from autophagy to apoptosis) [27, 122–124] or under certain conditions may induce cytoprotective autophagy [25, 125]. Recently, it has been demonstrated that stimulation of de novo ceramide synthesis results in dissociation of the complex formed between Beclin-1 and Bcl-2 through stimulating the phosphorylation of Bcl-2 by JNK1 leading to autophagy activation [122]. Likewise, inhibition of ceramide synthesis resulted in suppression of autophagy [122]. Ceramide has been reported to suppress the Akt signaling pathway resulting in autophagy activation via negative regulation of mTOR signaling [126], suggesting an upstream regulatory target of the autophagy pathway. Furthermore, ceramide-induced autophagy, either by targeting the mitochondria or by upregulation of Beclin-1, has been reported to be linked to autophagic cell death [27, 126, 127]. The role of ceramide-induced autophagy in the context of the ‘sphingolipid rheostat’ has yet to be fully understood and further investigation is required to determine the mechanisms involved in determination of cell fate when autophagy is activated.

S1P-induced autophagy may be associated with cell survival [41], consistent with the well-established ‘sphingolipid rheostat’. Lavieu et al. [41] reported that SphK1 (S1P)-induced autophagy protects the cell during nutrient starvation and cell death. Other sphingolipids such as gangliosides have been reported to induce the formation of autophagic vacuoles followed by cell death that is inhibited by either knockdown of autophagy genes or by an inhibitor of autophagy [128]. Thus, gangliosides appear to have a similar role in autophagic cell death as ceramides. It has been hypothesized that the structural properties of sphingolipids may mediate autophagosome formation and maturation [129]. In support of this hypothesis, inhibition of sphingolipid synthesis in *Saccharomyces cerevisiae* reduced autophagic activity, but had no effect on Atg12–Atg5 or Atg8-PE conjugation, or pre-autophagosomal structure formation [130]. This suggests that sphingolipids play a critical role in the formation of the autophagosome. Also, ceramides have been found in the membrane of the autophagosome, in line with the idea of a structural role of sphingolipids in autophagy [131]. This review will further discuss sphingolipid signaling and the consequences of their induced pathways on cell fate and the delicate balance between a healthy and disease state lung.

## Sphingolipids in the lung

### Significance of sphingolipids in lung development

It is widely understood that sphingolipids play key roles in regulating cellular homeostasis. However, their role in the regulation of lung development has been of particular interest and has received more attention. Ceramide, S1P, and their dynamic relationship have been of particular interest. The de novo pathway of ceramide synthesis has been linked to apoptotic endothelial cell death and decreased pulmonary barrier function [19, 132]. In a study conducted by Medler et al. [132], it was found that de novo ceramide synthesis resulted in apoptotic cell death in lung endothelial cells by both paracellular and TNF-α-stimulated intracellular ceramide signaling. Increased ceramide synthesis has also been linked to the development of emphysema-like disease states in the murine lung, suggesting that the balance between a pro-apoptotic molecule and a pro-survival factor, such as S1P, is essential in maintaining homeostasis in the vasculature of the lung [133]. Furthermore, investigation by Petrache et al. into ceramide-induced effects in the lung have revealed that an excess of ceramide-induced oxidative stress triggered apoptotic cell death which led to alveolar enlargement [134]. Superoxide dismutase has been shown to play a protective role against apoptosis and alveolar enlargement induced by disproportionate ceramide, suggesting that there are protective mechanisms that can be activated to protect or prevent unwarranted cell death in the healthy lung [134].

It is important to emphasize however, that a regulated level of ceramide may be necessary for maintenance of homeostasis in the lung. It is evident now that the preservation of the sphingolipid rheostat is important in the formation of lung structures at all stages of lung development and physiology [119]. A study showed that pigs exposed to fumonisin (FB_1_) (inhibitor of ceramide synthase) resulted in elevated levels of sphingolipids including sphinganine and sphingosine, which resulted in alveolar endothelial cell damage and fatal pulmonary edema [135]. Chronic exposure to FB_1_ in rats not only induced renal tumors, but also resulted in metastatic invasion of the lungs [136]. These findings suggest that ceramide synthesis is required for an underlying level of apoptotic cell death in the lungs as an anti-tumorigenic factor [136]. Xu et al. demonstrated that in murine lung epithelia the de novo ceramide synthesis pathway is the major contributing pathway for ceramide production [137]. Longevity assurance homolog 5 (LASS5) was found to be the predominant ceramide synthase in murine primary type II epithelial cells and SV40-transformed murine lung epithelial (MLE) cells. Indeed, inhibition of ceramide synthase activity by FB_1_ was shown to inhibit the production of ceramide [137]. Furthermore, this study also showed that overexpression of LASS5 reduced phosphatidylcholine (PtdCho) production, suggesting that ceramide may regulate PtdCho metabolism in pulmonary cells [137].

In-depth investigation into the levels of sphingolipids in a developing rat lung revealed that maintenance of sphingolipid levels and their metabolism are highly regulated. In the embryonic rat lung, sphingomyelin and sphingosine levels increase during development and plateau at birth. Interestingly, the activity profiles of SPT, aSMase and nSMase correlate accordingly [138]. In contrast to the rat lung, sphingomyelin levels in the fetal lung of the monkey and lamb decrease during development [139, 140]. These findings suggest that regulation of sphingolipid metabolism is important in proper lung development and any imbalances or misregulations in the sphingolipid rheostat could lead to improper lung structure or disease states (Table 1).

**Table 1:** Effects of sphingolipids in pulmonary diseases

Stimulus	Sphingolipid	Enzyme/pathway	Cell fate	Pathophysiological effect	Disease state	Reference
Sepsis, inhalation of harmful substances	↑ S1P	NSM pathway, ceramide is converted to S1P	↓ Neutrophil apoptosis	Expedites inflammation, increase in proteases and ROS resulting in damage to lung tissues	Acute lung injury/ARDS	[142]
Mechanical ventilation, high oxygen	↑ Ceramide	? (Possibly de novo synthesis pathway)	↑ Alveolar epithelial cell apoptosis	Halt in alveolarization, fewer and larger alveoli	BPD	[21, 118]
Airway response to allergen	↑ S1P	Mitogenic factor, facilitates G1/S progression	↑ Airway smooth muscle cell proliferation	Airway wall remodeling	Asthma	[161]
Genetic variation at the 17q21 locus (ORMDL3 protein)	↓ Ceramide	Inhibition of SPT	Increase of S1P, decrease of ceramide results in cell survival and proliferation	Genetic predisposition to play role in pathogenesis of asthma	Asthma	[159, 166, 167]
Inhalation of cigarette smoke/pollutants	↑ Ceramide	↑ De novo synthesis	↑ Alveolar epithelial cell apoptosis	Loss of alveolar surface area available for gas exchange	COPD/emphysema	[19]
Inhalation of cigarette smoke/pollutants	↑ Ceramide	?	↑ Impaired autophagy	Accumulation of impaired autophagy marker p62 in autophagosomes	COPD/emphysema	[184, 190]
*P. aeruginosa* infection in CFTR-deficient mice	↑ Ceramide	? (Possibly by a shift in balance of enzymes involved in ceramide metabolism)	↑ Bronchial cell apoptosis	Bronchial cell apoptosis and DNA deposition in the upper airways	Cystic fibrosis	[6]

? means currently unknown

### Sphingolipids in pulmonary disease

#### Acute lung injury/ARDS

Acute lung injury (ALI) and acute respiratory distress syndrome (ARDS) are conditions that are characterized by lung inflammation, increased microvascular permeability, and edema [119, 141]. The contributing role of sphingolipids in attenuating injury to the lung during ALI and ARDS has been recently explored and is now well established that there are a number of mechanisms including the neutral sphingomyelinase (nSMase), ceramide, S1P, and the p38 MAPK pathway, that appear to be involved in the pathological process [2, 142].

The influence that sphingolipids have on neutrophil cell fate in an ALI/ARDS disease state is of particular interest as it is well established that the longevity of neutrophils correlates with the severity of the pathological state [143, 144]. Under homeostatic conditions, neutrophils will undergo unprompted apoptosis [145]. However, neutrophils that are recruited to occupy the alveolar space during lung injury contribute to the pathogenesis of ALI and ARDS by production and secretion of proteases and ROS for the duration of their survival [146]. Thus, neutrophil apoptosis is essential for resolution of inflammation, especially in the context of an injury to the lung.

It has been reported that apoptosis of neutrophils from patients with sepsis was suppressed via a mechanism involving NF-κB, reduced caspase-9 and caspase-3 activity, and maintenance of mitochondrial membrane potential [147]. Another study found that sphingolactone-24 (Sph-24) (nSMase inhibitor) and SKI-II (sphingosine kinase inhibitor) prevented the anti-apoptotic effect of lipopoly saccharides (LPS) on neutrophils, and that LPS stimulates sphingomyelinase activity suggesting that sphingolipids are involved in inhibition of neutrophil apoptosis [142]. When Sph-24 was administered to LPS challenged lungs in mice, counts of leukocytes decreased after a period of 24 h suggesting that nSMase accelerates injury progression during lung injury by deceleration of neutrophil apoptosis [142]. They also demonstrated that LPS challenge of neutrophils that have been recruited to the lung also display an increase in S1P levels. This study also revealed that the increased levels of anti-apoptotic S1P are likely the result of activation of the nSMase-S1P pathway [142]. S1P is generated by SphKs that are stimulated by factors such as histamines and cytokines which could explain the increase of SphK activity in the injured lung [142, 148]. Thus, both nSMase and S1P play key roles in the pathogenic process of ALI/ARDS, particularly in their influence of preventing neutrophil apoptosis. In ALI, increased nSMase activity augments ceramide and subsequent sphingosine formation that then is phosphorylated to S1P by activated sphingosine kinase, leading to phosphorylation of p38 MAPK resulting in inhibition of neutrophil apoptosis [142, 149]. Greater understanding in the pathogenesis of ALI/ARDS and the role sphingolipids play in cell fate will improve detection and treatment of this critical condition.

#### Bronchopulmonary dysplasia

Bronchopulmonary dysplasia (BPD) is amongst the most common chronic lung diseases of neonates affecting between 52 and 7 % of infants born weighing between 500 and 1500 g, respectively [150]. Very premature infants that are subjected to mechanical ventilation and oxygen supplementation due to respiratory failure are prone to lung injury that may result in chronic lung disease, such as BPD, with lifelong consequences [151]. The lungs of present day BPD patients are characterized by a halt in lung development, with simplification of normal lung complexity as seen with fewer and larger alveoli and vascular abnormalities [152]. It was recently demonstrated that changes in sphingolipid levels may affect proper lung development and function when Tibboel et al. elegantly demonstrated that newborn mice that were exposed to hyperoxic conditions displayed an increase in multiple sphingolipids, including ceramide [118]. This caused abnormal alveolar morphology and obstructive lung function that were only partially recovered in room air [118]. The use of D-sphingosine reduced ceramide levels and partially minimized halt in alveolarization, demonstrating that altered sphingolipid levels may be a factor in hyperoxia-induced lung injury or BPD [118]. Recently, it was shown that levels of vascular endothelial growth factor (VEGF), a critical factor in pulmonary vascular development, were decreased in a rodent animal model of BPD [153], thereby most likely reducing alveolarization [154, 155]. Increased ceramide production has also been shown to decrease VEGF by suppression of hypoxia-inducible factor-1α [156]. These findings suggest that sphingolipids play a role in abnormal lung vascular development through VEGF signaling in BPD. In another study, apoptosis was detected in alveolar epithelial cells in the lungs of preterm infants that were subjected to ventilation and oxygen treatment [157]. Similar results have been reported in mouse models of BPD [158]. Kroon et al. observed that mechanical ventilation of newborn rat resulted in increased number of apoptotic alveolar type II cells [21]. Prolonged maximal cyclic stretch was also associated with increased expression of cleaved caspase-3, -7, and -8, as well as apoptotic mediator Fas ligand (FasL), suggesting that the extrinsic death pathway via the FasL/Fas system is involved in ventilation-induced apoptosis of alveolar type II cells [21].Given these findings, it is worthwhile to investigate the role of ventilation-induced ceramide production in epithelial cell apoptosis as a mechanism responsible for pulmonary apoptosis and inhibition of alveolar development in preterm infants with BPD. The possibility that altered levels of various sphingolipids can change cell fate during mechanical ventilation opens potential areas of therapeutic interventions for BPD to reduce lung damage in premature infants that require respiratory assistance.

#### Asthma

Asthma is a complex chronic inflammatory lung disease that is characterized by airway wall remodeling, airway smooth muscle contraction and hyperreactivity, increased mucus production, and inflammatory cell gathering [159]. It has increasingly become apparent that sphingolipid metabolites play a regulatory role in the pathogenesis of asthma [160]. S1P is often attributed to act as a pro-survival signal in cells [9], and increased levels in the asthmatic airway appear to play a role in perpetuating the asthmatic response [161]. The role of S1P in the pathogenesis of asthma is well illustrated by the fact that S1P is involved in trafficking and chemotaxis of immune cells, such as mast cells, to the airway. Interestingly, S1P release from the immune cell and binding (in autocrine and paracrine signaling) with S1P_1_ receptors in low antigen concentrations facilitates mast cell chemotaxis, while activation of S1P_2_ receptors in high antigen concentrations ceases chemotaxis and initiates degranulation [162]. S1P not only plays an important role in immune cell movement, but also appears to act as a mitogenic factor and a stimulator of proliferation of the airway smooth muscle cells. S1P increases G_1_/S progression in the cell cycle which results in augmented EGF- and thrombin-induced DNA proliferation [161, 163]. SphK has been shown to play important roles in other inflammatory and hyper proliferative diseases, and has received attention with the prospect of developing specific SphK inhibitors for therapeutic and treatment purposes [164].

Research efforts have recently been focused on the possibility that genetic predisposition of the asthmatic respiratory tract significantly affects the severe reaction to allergic and/or inflammatory stimuli with strong evidence that genetic variation at the 17q21 locus is linked with childhood asthma [165]. The locus gene orosomucoid 1-like 3 (*ORMDL3*) that belongs to a gene family encoding ER transmembrane proteins has been recently identified to be related to the pathogenesis of asthma [166, 167]. Systematic evaluation of single nucleotide polymorphisms (SNPs) and patients with childhood onset asthma have revealed that genetic variants regulating *ORMDL3* expression may alter susceptibility to asthma [167].Orosomucoid-like (ORMDL) proteins act as negative regulators of SPT that are mediated by a feedback response, thus increased levels of the ORMDL proteins result in a decrease in ceramide and higher order sphingolipid biosynthesis as a result of inhibited *de novo* sphingolipid synthesis [168, 169]. Expression of the *ORMDL3* gene is increased in asthmatic airways, which results in decreased de novo ceramide synthesis and also has been associated with airway hyperreactivity in mouse lungs, and human and murine bronchial rings [159]. Worgall et al. [159] investigated the effect of decreased SPT activity in the lung by pharmacologic intervention with myriocin (SPT inhibitor) as well as in SPT heterozygous knockout mice and observed increased bronchial reactivity in the absence of inflammation. They also found that intracellular magnesium homeostasis and bronchial sensitivity to magnesium was affected by decreased SPT activity, providing a mechanistic link between decreased de novo sphingolipid synthesis, altered contractile sensitivity to magnesium, and smooth muscle function in asthma [159]. This emerging evidence that ORMDL proteins affect sphingolipid metabolism in asthmatic airways suggests that it is highly likely that a genetic predisposition to altered sphingolipid metabolism plays a significant role in the pathogenesis of asthma.

#### COPD and emphysema

Chronic obstructive pulmonary disease (COPD) encompasses a spectrum of pathological conditions, including emphysema, that are characterized by difficulties breathing due to obstructed airflow [170]. Emphysema is found almost exclusively in adults and is often associated with inhalation of cigarette smoke but can also result from exposure to environmental pollutants, typically worsening over time [171]. Patients with emphysema particularly have a loss of alveolar surface area available for gas exchange due to destruction of distal airway spaces [171]. The mechanisms involved in the pathogenesis of COPD include inflammatory responses in the airway, loss of barrier function, oxidative, and ER stress responses. In addition, both apoptosis and autophagy of the cells in the airways are generally accepted to be important events in the pathogenesis of COPD and pulmonary emphysema [172]. However, it is very important to note that in most studies the smoking status of the control population was unknown or was significantly different from that of the COPD subjects [173–176]; thus, it is not fully understood whether the habit of smoking has a role in inducing apoptosis independently of airflow limitation.

It is now recognized that sphingolipids play important roles in the development of COPD with no surprise that ceramide appears to be a crucial mediator in the apoptotic death of alveolar cells [19, 177]. For instance, in rat and mice emphysema models, inhibition of de novo synthesis of ceramide induced by VEGF blockade prevented alveolar cell apoptosis and oxidative stress [19]. Moreover, when ceramide was introduced through intratracheal instillation of mice, apoptosis of lung alveolar cells and total ceramide levels increased [19]. More importantly, lung ceramide levels are markedly higher in individuals with emphysema from chronic cigarette smoking compared to individuals without emphysema [19]. In this study, the authors conclude that de novo ceramide synthesis is essential for the development of murine lung emphysema [19]. At the molecular level, activation of nSMase2 during cigarette smoke-induced oxidative stress of human airway epithelial (A549) cells has been shown to generate ceramide and apoptosis [178]. Both proto-oncogene tyrosine-protein kinase *Src* and p38 MAPK appear to be involved in regulating nSMase2 phosphorylation and activation [179].

Despite higher expression of ceramide in the lungs of COPD and idiopathic pulmonary fibrosis (IPF) patients, this phenomenon does not appear to be specific and related to COPD severity [180]. Scarpa and colleagues [180] studied 10 subjects with severe COPD, 13 with mild/moderate COPD, 11 with IPF, 12 non-COPD smokers, and 11 nonsmoking controls. They found an increase in ceramide levels in COPD patients compared to control smokers, which correlated to the impairment of gas exchange but not to the degree of airflow limitation. A recent study conducted by Tibboel et al. revealed that the addition of a SPT inhibitor to a rodent model of elastase-induced emphysema diminished the increase in ceramides and improved lung function [177] suggesting that ceramide upregulation in emphysema models may be a critical factor in the development of alveolar destruction in the disease state, and serves as a potential therapeutic target that warrants further investigation.

The functional significance of autophagy in disease states such as COPD has yet to be established since relatively few studies have been done on the lung. Few in vivo and in vitro specimens of COPD lung exhibit increased levels of autophagy markers when exposed to cigarette smoke or cigarette smoke extract, possibly as a response to a source of stress on the lung [73, 128, 181], and there have been reports suggesting that the autophagic process plays an important role in the pathogenesis of COPD perhaps via promoting epithelial cell death [181–183]. Recently, Fujii et al. found autophagy impairment in COPD [184]. Despite high baseline levels of autophagy in primary human bronchial epithelial cells isolated from COPD patients, cigarette smoke extract significantly reduced autophagy [184]. Furthermore, higher levels of p62 and ubiquitinated proteins have been detected in lung homogenates from COPD patients suggesting that insufficient autophagic clearance of damaged proteins may be involved in cell senescence in COPD patients [184]. As mentioned earlier, increased expression of ceramide has been detected in emphysema and may play a critical role in impaired autophagy in the context of COPD. However, the role of ceramide in smoke-induced COPD is not yet well studied and it may be worthwhile to investigate whether the increase of ceramide content in lungs when exposed to cigarette smoke also has an impact on autophagy, and if the pathway plays a pathogenic role in COPD.

#### Cystic fibrosis

Cystic fibrosis(CF) is caused by mutations in the cystic fibrosis transmembrane conductance regulator (*CFTR*) gene that codes for a chloride channel that mediates proper movement of chloride ions in epithelial cells [185, 186]. The disease has a median predicted age of survival of 49.7 years [187] and is characterized by accumulation of mucus in the bronchi, and reduced mucociliary clearance ultimately leading to recurrent and/or chronic infections with bacteria including *S. aureus* and *P. aeruginosa* [162]. Ceramide has been measured at higher concentrations in CF airways and appears to be an important factor in the hallmark features of CF and cellular fate in the affected lungs [6]. Several studies have shown that CFTR dysfunction leads to an imbalance in sphingolipid homeostasis [6] and that its localization in the lipid raft influences membrane ceramide composition [188, 189]. Furthermore, results of studies using *Cftr* ^−*/*−^ mice and human lung tissue demonstrate that CFTR expression inversely correlates with ceramide accumulation and severity of emphysema in COPD subjects, demonstrating a critical role of membrane-localized CFTR in ceramide regulation and inflammation in lung injury and emphysema [188, 189]. Ceramide appears to be a critical regulator of *P. aeruginosa* infection in CFTR-deficient mice [6]. CFTR-deficiency results in alkalinization of acidic prelysosomes and lysosome vesicles which likely alter the balance of enzymes that increase and decrease levels of ceramide in the cells, which ultimately results in an increase of ceramide concentration in the lung epithelial cells of CFTR-deficient mice [6]. Accumulation of ceramide in the cells led to apoptosis and DNA deposition in the upper airways that facilitated *P. aeruginosa* infection. Treatment of CFTR-deficient mice with amitriptyline (aSMase inhibitor) normalized ceramide levels and prevented pulmonary infection with *P. aeruginosa* [6]. CFTR-deficient mice also display an upregulation and activation of CD95 (Fas/APO-I receptor) caused by increased ceramide concentrations, further propelling in a feedback cycle [185]. CD95 is also involved in an increase of bronchial cell death, identifying a potential relationship between CD95 and ceramide as an important mechanistic link in CF [185]. It has yet to be fully understood if these findings can be transferred into a human system, however results thus far have suggested that disruption of ceramide formation may serve as a prospective treatment for patients with CF.

## Closing remarks

Sphingolipids play an important role in many physiological and pathological processes across many organ systems and diseases. The vast number of sphingolipids is reflected in the spectrum of signaling mechanisms they are involved in. Different sphingolipids have key roles in determining cell fate in many cells throughout nearly all organ systems. Ceramide is known to induce apoptosis in cells while S1P counterbalances ceramide and promotes cell survival in what is referred to as the sphingolipid ‘rheostat’. These effects are of significance in the lungs and its diseases such as ALI/ARDS, BPD, asthma, COPD, emphysema, and CF. Investigation into the lung, its diseases, and the effects of sphingolipids on cell fate using animal and human models have provided great insight into the pathogenesis of the disease states as well as potential interventions that can be used to improve outcomes for patients. In this regard, inhibitors of the sphingolipid pathway show great therapeutic potential and should be investigated and functionally validated for their protective effects and benefit for the patients. This certainly broadens the options of treatment and care for those suffering from devastating lung diseases for which treatments are limited.

## Abbreviations

ALI: Acute lung injury
AMPK: AMP-activated protein kinase
Apaf-1: Apoptotic protease-activating factor-1
ARDS: Acute respiratory distress syndrome
aSMase: Acid sphingomyelinase
ATG: Autophagy related genes
BPD: Bronchopulmonary dysplasia
Caspase: Cysteine aspartate-specific protease
CF: Cystic fibrosis
CFTR: Cystic fibrosis transmembrane conductance regulator
CMA: Chaperone-mediated autophagy
COPD: Chronic obstructive pulmonary disease
DAPK: Death-associated protein kinase
DD: Death domains
DISC: Death-inducing signaling complex
ER: Endoplasmic reticulum
FasL: Fas ligand
FB1: Fumonisin B1
IPF: Idiopathic pulmonary fibrosis
JNK1: c-Jun NH2–terminal kinase 1
LASS5: Longevity assurance homolog 5
LC3: Microtubule-associated protein 1 light chain 3
LPS: Lipopolysaccharides
MCRM: Mitochondrial ceramide-rich macrodomain
MLE: Murine lung epithelial
MOMP: Mitochondrial outer membrane permeabilization
mTOR: Mammalian target of rapamycin
mTORC1: mTOR complex 1
NF-κB: Nuclear factor-κB
nSMase: Neutral sphingomyelinase
ORMDL: Orosomucoid-Like
PCD: Programmed cell death
PE: Phosphatidylethanolamine
PI3K: Phosphatidylinositol 3-kinase
PI3P: Phosphatidylinositol-3-phosphate
PKCζ: Protein kinase C ζ
PP2A: Protein phosphatase 2
PtdCho: Phosphatidylcholine
RIP: Receptor interacting protein
ROS: Reactive oxygen species
S1P: Sphingosine-1-phosphate
SMase: Sphingomyelinase
SNP: Single nucleotide polymorphism
Sph-24: Sphingolactone-24
SphK: Sphingosine kinase
SPT: Serine palmitoyltransferase
tBID: Truncated BID
TNF: Tumor necrosis factor
TRAIL: TNF-related apoptosis-inducing ligand
ULK1: UNC-51-like kinase
UPR: Unfolded protein response
VEGF: Vascular endothelial growth factor
